# Intron-Dependent or Independent Pseudouridylation of Precursor tRNA Containing Atypical Introns in *Cyanidioschyzon merolae*

**DOI:** 10.3390/ijms232012058

**Published:** 2022-10-11

**Authors:** Yasuha Nagato, Chie Tomikawa, Hideyuki Yamaji, Akiko Soma, Kazuyuki Takai

**Affiliations:** 1 Department of Materials Science and Biotechnology, Graduate School of Science and Engineering, Ehime University, Matsuyama 790-8577, Ehime, Japan; 2 Graduate School of Horticulture, Chiba University, Matsudo 271-8510, Chiba, Japan

**Keywords:** tRNA modification, pseudouridine, multiple-sites-specific enzyme, tRNA processing

## Abstract

Eukaryotic precursor tRNAs (pre-tRNAs) often have an intron between positions 37 and 38 of the anticodon loop. However, atypical introns are found in some eukaryotes and archaea. In an early-diverged red alga *Cyanidioschyzon merolae*, the tRNA^Ile^(UAU) gene contains three intron coding regions, located in the D-, anticodon, and T-arms. In this study, we focused on the relationship between the intron removal and formation of pseudouridine (Ψ), one of the most universally modified nucleosides. It had been reported that yeast Pus1 is a multiple-site-specific enzyme that synthesizes Ψ34 and Ψ36 in tRNA^Ile^(UAU) in an intron-dependent manner. Unexpectedly, our biochemical experiments showed that the *C. merolae* ortholog of Pus1 pseudouridylated an intronless tRNA^Ile^(UAU) and that the modification position was determined to be 55 which is the target of Pus4 but not Pus1 in yeast. Furthermore, unlike yeast Pus1, cmPus1 mediates Ψ modification at positions 34, 36, and/or 55 only in some specific intron-containing pre-tRNA^Ile^(UAU) variants. cmPus4 was confirmed to be a single-site-specific enzyme that only converts U55 to Ψ, in a similar manner to yeast Pus4. cmPus4 did not catalyze the pseudouridine formation in pre-tRNAs containing an intron in the T-arm.

## 1. Introduction

Eukaryotic transfer RNA (tRNA) genes are transcribed by RNA polymerase III, and the transcript then goes through a complex maturation pathway that involves trimming of the 5′ and 3′ ends, the addition of a CCA terminus, removal of intronic sequences, and modification of multiple nucleosides [1]. The order of these processes, in particular, that of intron removal and nucleoside modification, is variable among different tRNA genes and specific nucleoside modifications [2]. Modified nucleosides that are insensitive to introns include dihydrouridine at position 17 (D17), *N*^2^,*N*^2^-dimethylguanosine at position 26 (m^2^_2_G26), pseudouridine at position 39 (Ψ39), 7-methylguanosine at position 46 (m^7^G46), 5-methylcytidine at position 49 (m^5^C49), 5-methyluridine at position 54 (m^5^U54), and Ψ55 [3,4].

Some other modified nucleosides are introduced after splicing [5]. For example, in an in vitro experiment using a cell-free splicing system with an intron-containing precursor tRNA (pre-tRNA) specific for Tyr from tobacco, m^1^G37 and Ψ39 were detected only in mature tRNA^Tyr^ [5]. Other nucleosides, such as 2′-*O*-methylguanosine at position 18 (Gm18), 2′-*O*-methylcytidine at position 32 (Cm32), Gm34, Ψ32, 1-methylguanosine at position 37 (m^1^G37), wybutosine at position 37 (yW37), *N*^6^-(isopentenyl)adenosine at position 37 (i^6^A37), and 2′-*O*-methyluridine at position 44 (Um44) have also been shown to be introduced after splicing [3,6,7,8,9].

In the most notable cases, tRNA modifications are introduced prior to the removal of the intron and added to a specific tRNA processing intermediate [6,10]. For example, the *S. cerevisiae* amber suppressor tRNA^Leu^(CUA) gene encodes an intron in the anticodon arm, and the intronless tRNA^Leu^ mutant does not have the 5-methylcytidine modification at position 34 (m^5^C34) and position 40 (m^5^C40) [3,11]. Moreover, the deletion of the intron from the yeast suppressor tRNA^Tyr^ gene causes a decrease in the suppressor activity, due to the absence of Ψ at position 35 [7]. Moreover, in the case of plant pre-tRNA^Tyr^, the specific intron sequence is important for the Ψ35 introduction [12]. 

Ψ is formed by the C5-ribosyl isomerization of U by pseudouridine synthetases and is found in quite various positions in tRNA [13]. In yeast pre-tRNA^Ile^(UAU), which contains an intron in the anticodon arm, Ψ modifications are introduced at positions 27, 34, 36, 55, and 67 [14]. Ψ27, Ψ34, Ψ36, and Ψ67 are synthesized by Pus1 while Ψ55 is formed by Pus4 [15,16]. Pus1 is a multiple-sites-specific enzyme that also forms Ψ1, Ψ26, Ψ28, Ψ35, and Ψ65 in other tRNA species [15,17,18]. In contrast, Pus4 is a single site-specific pseudouridine synthase that mediates the formation of Ψ only at position 55. Ψ34 and Ψ36 are intron-dependent (requiring) modifications, whereas Ψ55 is insensitive to the presence of an intron [3,19].

**Figure 1:**
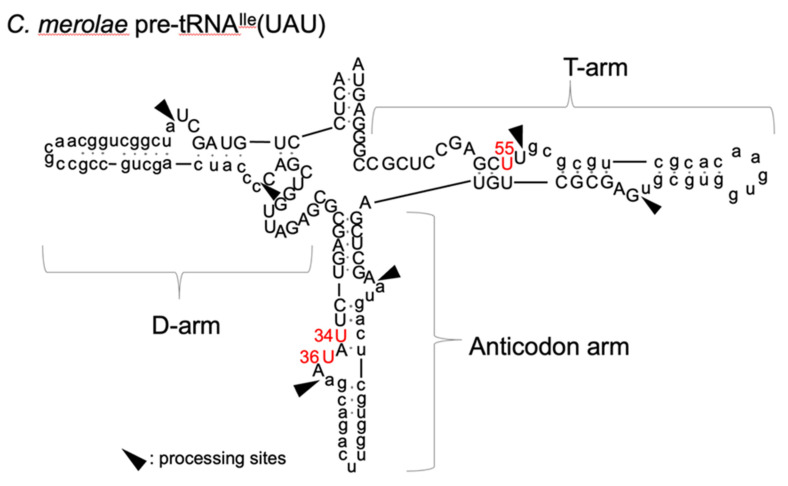
An inferred secondary structure of pre-tRNA^Ile^(UAU) of *C. merolae*. Three introns, shown in lowercase letters, interrupt D-arm, anticodon arm, and T-arm of the mature tRNA^Ile^(UAU) nucleotide, shown in uppercase letters. In yeast tRNA^Ile^(UAU), U34/U36 and U55 are pseudouridylated by yeast Pus1 and Pus4, respectively. U34, U36, and U55, which may be pseudouridylated by cmPus1 and cmPus4, are indicated in red.

In the present work, we aimed at clarifying the relationship between introns and the Ψ synthesis in tRNA^Ile^(UAU), in *Cyanidioschyzon merolae*, an acidothermophilic red alga that inhabits an extreme environment (pH1-3, 40–50 °C) [20]. *C. merolae* is characterized by its unusual organization of tRNA genes: Permuted tRNA genes are found in this organism, in which the 3′-half of the tRNA coding sequence is upstream of the 5′-half in the genome [21]. Furthermore, of all nuclear tRNA genes in *C. merolae*, over 62% of genes contain an intron coding region(s). This proportion is higher than in other eukaryotes, such as *S. cerevisiae* (20%), human (5%) and *Arabidopsis thaliana* (13%) (http://lowelab.ucsc.edu/GtRNAdb/ accessed on 1 October 2021) [22]. Multiple intron coding regions are found in no less than 12 tRNA genes of *C. merolae* [23] out of which three, including the tRNA^Ile^(UAU) gene, encode three introns in the D-, anticodon, and T-arm regions (Figure 1). While the anticodon arm intron in pre-tRNA^Ile^(UAU) is removed after processing the other two introns, nothing more is known regarding the processing mechanism. This raises the question of what happens to the relationship between the introns and nucleoside modifications in this organism. To address this question, Ψ is a good target because the mature tRNA^Ile^(UAU) is very likely to be modified at least at 34, 36, and 55 as yeast tRNA^Ile^(ΨAΨ) is. While the positions of Ψ modifications on *C. merolae* tRNA^Ile^(UAU) have not been determined, the organism has the ortholog proteins of Pus1 (cmPus1) and Pus4 (cmPus4), which are expected to modify, by analogy from the yeast case, positions 34 and 36 and position 55, respectively.

In this article, we first demonstrate that cmPus1 unexpectedly mediates Ψ modification in intronless tRNA^Ile^(UAU), and that the Ψ modification was at 55 instead of being at 34 and 36. To some pre-tRNA^Ile^(UAU) variants that contain one or more intron(s), cmPus1 introduced Ψ at positions 34, 36, and 55, but not to the others. cmPus4 converted U55 to Ψ55 in intron-less and -containing tRNA^Ile^(UAU), but not in pre-tRNA^Ile^(UAU) containing an intron in the T-arm. All these demonstrate a novel relationship between tRNA introns and the Ψ syntheses.

## 2. Results

### 2.1. Inferred Secondary Structure of Pre-tRNA^Ile^(UAU) from C. merolae

Figure 1 shows an inferred secondary structure of pre-tRNA^Ile^(UAU) from *C. merolae* [23]. This pre-tRNA contains three introns in the D-arm, anticodon-loop, and T-arm. U34, U36, and U55, which would be pseudouridylated by yeast Pus1 and Pus4, are indicated in red. Pseudouridylations of U34 and U36 by Pus1 are intron-dependent, and Ψ55 synthesized by Pus4 is insensitive to the presence of an intron in yeast [3,19]. By what mechanisms do cmPus1 and cmPus4 act on the pre-tRNA species, and how does the presence or absence of the intron affect the activity of cmPus1 and cmPus4?

### 2.2. Analysis of Pseudouridine Formation in Intronless tRNA^Ile^(UAU) by cmPus1 Candidate 

**Figure 2:**
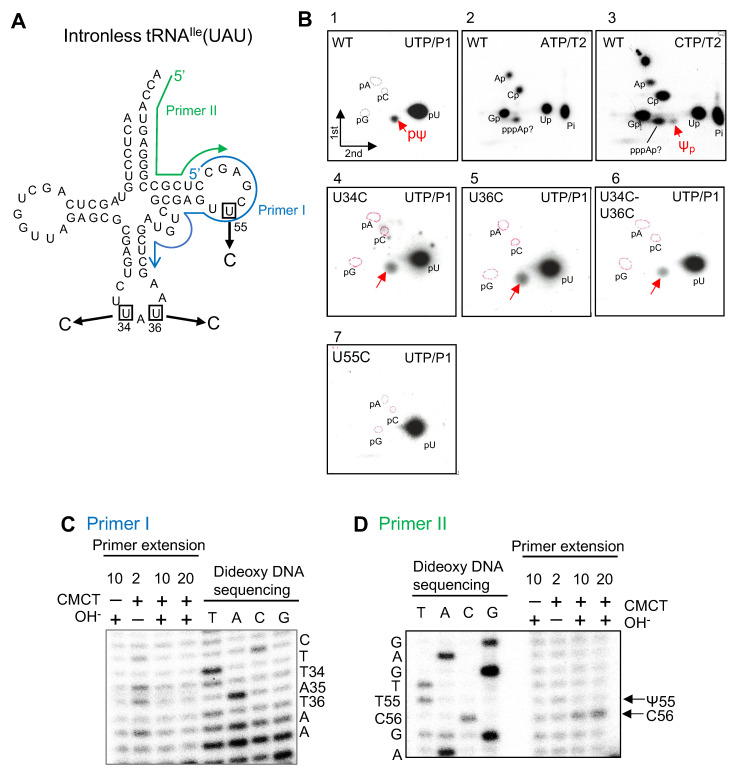
cmPus1 pseudouridylates the intronless tRNA^Ile^(UAU) transcript in vitro. (**A**) The cloverleaf structure of intronless tRNA^Ile^(UAU) of *C. merolae* is shown. Mutation positions are indicated by squares on the cloverleaf structure of intronless tRNA^Ile^(UAU) of *C. merolae*. The regions complementary to the primers used for reverse transcription are marked as a blue arrow (primer I, position 60 to 40) and a green arrow (primer II, position 76 to 59) on the cloverleaf schematic. (**B**) UTP, CTP, and ATP refer to the ^32^P-labeled nucleotide incorporated during the transcription reaction. The ^32^P-internally labeled wild type (WT) or each mutant (U34C, U36C, U34C-U36C, or U55C) of the tRNA^Ile^(UAU) transcript was incubated with cmPus1, and then the RNA was digested by nuclease P1 (P1) or RNase T2 (T2). Ψ formation was analyzed by 2D-TLC. Spots derived from pΨ and Ψp are indicated by red arrows. Positions of standard markers (pA, pG, and pC) are enclosed by red dotted circles. (**C**,**D**) A complementary primer as shown with the primer I blue (**C**) or primer II green (**D**) arrow in A was used for reverse transcription. Primer extensions were performed on the wild type tRNA^Ile^(UAU) transcripts treated with cmPus1. The transcripts were treated with CMCT for 2, 10, or 20 min plus or minus alkaline (OH^−^) treatment (+ or −). Dideoxy sequencing was performed with the same primer.

Initially, we investigated whether purified cmPus1 possesses pseudouridine synthase activity. Internally ^32^P-UTP-labeled tRNA^Ile^(UAU) transcript, in which introns were not included (Figure 2A), was treated with purified cmPus1 (Figure S1A). Because it was reported that yeast Pus1 did not synthesize Ψ into mature size tRNA without an intron [15], we performed this experiment as a negative control. Unexpectedly, however, 2D-TLC analysis of modified nucleotides in this sample showed the formation of ^32^P-labeled pΨ (Figure 2B, panel 1). Thus, this result reveals that the substrate RNA specificity of cmPus1 is completely different from that of yeast Pus1. 

We also performed a nearest neighbor analysis. Intronless tRNA^Ile^(UAU) transcript was ^32^P-labeled using α-^32^P-CTP or α-^32^P-ATP, and then the labeled tRNA was incubated with purified cmPus1. The modified tRNA was digested with RNase T2 (Figure 2B, panels 2 and 3). Ψp was only detected when the tRNA was CTP-labeled (Figure 2B, panels 3). These results indicate that the Ψ formation occurs 5′-adjacent to cytidine and are limited to the positions at U3, U12, U31, U41 U47, U55, and U62 in the intronless tRNA^Ile^(UAU).

Furthermore, we investigated Ψ formation using ^32^P-UTP-labeled-mutant tRNAs in which U at positions 34 and/or 36 was substituted with C because C is not subject to the Ψ formation (Figure 2B, panels 4–6). The result of the 2D-TLC showed that Ψ formations occurred in both the tRNA^Ile^ U34C and U36C mutants (Figure 2B, panels 4 and 5). At the start of this experiment, we predicted that cmPus1 would produce Ψ modifications at positions 34 and 36 in intronless tRNA^Ile^(UAU). However, Ψ formation could also be detected in the tRNA^Ile^ U34C-U36C double mutant (Figure 2B, panel 6). On the contrary, 2D-TLC analysis using tRNA^Ile^ U55C mutants showed that cmPus1 did not mediate the formation of Ψ (Figure 2B, panel 7). Moreover, we monitored the pseudouridylation activity of cmPus1 against ^32^P-UTP-labeled-tRNA^Ile^ U34C-U55C, U36C-U55C, and U34C-U36C-U55C mutants. The pΨ spot was not observed in these samples (Figure S2). 

In addition, the modification position(s) was analyzed by a combination of 1-cyclohexyl-3-(2-morpholinoethyl) carbodiimide metho-p-toluene sulfonate (CMCT)-chemical modification and primer extension [24]. We performed the reverse transcription with tRNA samples treated with cmPus1 using a DNA primer complementary to the sequence from position 60 to 40 (Primer I) or 76 to 59 (Primer II) of tRNA^Ile^(UAU) (Figure 2A and Table S1). Alkaline incubation allows the hydrolysis of U and Ψ adducts, whereas Ψ-CMC adducts are insensitive, and Ψ-CMC stops reverse transcription. Thus, reverse transcription stops indicate the position of Ψ-CMC. Guanosine is also modified with CMCT, but less efficiently than U and Ψ, and the G adduct is easily removed by the alkaline treatment. No stops in the reverse transcription with primer I at either position 34 or 36 were observed (Figure 2C). On the other hand, the reverse transcription with primer II stopped at position 56 (Figure 2D), and the Ψ position was predicted to be at 55 but not at 34 and 36. These results are consistent with the 2D-TLC results. At least in our in vitro experiment, pseudouridylation by cmPus1 occurs at position 55 and not at positions 34 and 36 in intronless tRNA^Ile^(UAU).

To investigate whether the Ψ formation at position 55 occurs only in tRNA^Ile^(UAU), we also analyzed cmPus1 activity for tRNA^Ile^(GAU), which possesses an intron in the anticodon-loop (Figure S3A). Although Ψ formations were detected in both the intronless and intron-containing tRNA^Ile^(GAU), the activities diminished in the U55C mutant (Figure S3B). Thus, the pseudouridylation at position U55 by cmPus1 is not limited to tRNA^Ile^(UAU).

### 2.3. CmPus4 Possesses a tRNA Ψ55 Synthase Activity

**Figure 3:**
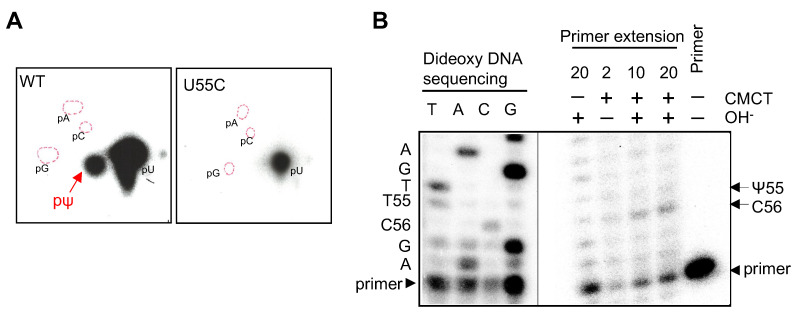
Pseudouridylation activities of cmPus4 towards intronless tRNA^Ile^(UAU) in vitro. (**A**) The α-^32^P-UTP-labeled tRNA^Ile^(UAU) transcript of the wild type or the U55C mutant was incubated with cmPus4, and the Ψ formation was analyzed by 2D-TLC. A pΨ spot was detected only in the wild type tRNA^Ile^(UAU) transcript. (**B**) The position of Ψ in intronless tRNA^Ile^(UAU) treated with cmPus4 was detected by CMCT-RT using the primer II as shown in Figure 2A. The transcripts were treated with CMCT for 10 or 20 min plus or minus alkaline (OH^−^) treatment (+ or −). Ψ modification was detected at position 55.

Our experimental results show that cmPus1 possesses a tRNA Ψ55 synthase activity. In yeast, Ψ55 is synthesized by Pus4. Therefore, we investigated the activity of cmPus4. As with cmPus1, wild type and the U55C mutant tRNA^Ile^(UAU) transcripts were treated with purified cmPus4 (Figure S1B). The modified nucleotides were analyzed by 2D-TLC, and pΨ was detected in the wild type tRNA sample (Figure 3A, left panel). In contrast, pΨ formation was not observed in the U55C mutant tRNA^Ile^(UAU) transcript (Figure 3A, right panel). This result shows that cmPus4 possesses a tRNA Ψ55 synthase activity, such as yeast Pus4. The results of primer extension confirmed this conclusion (Figure 3B).

### 2.4. Analysis of Pseudouridine Formation in Pre-tRNA^Ile^(UAU) by cmPus1 In Vitro 

**Figure 4:**
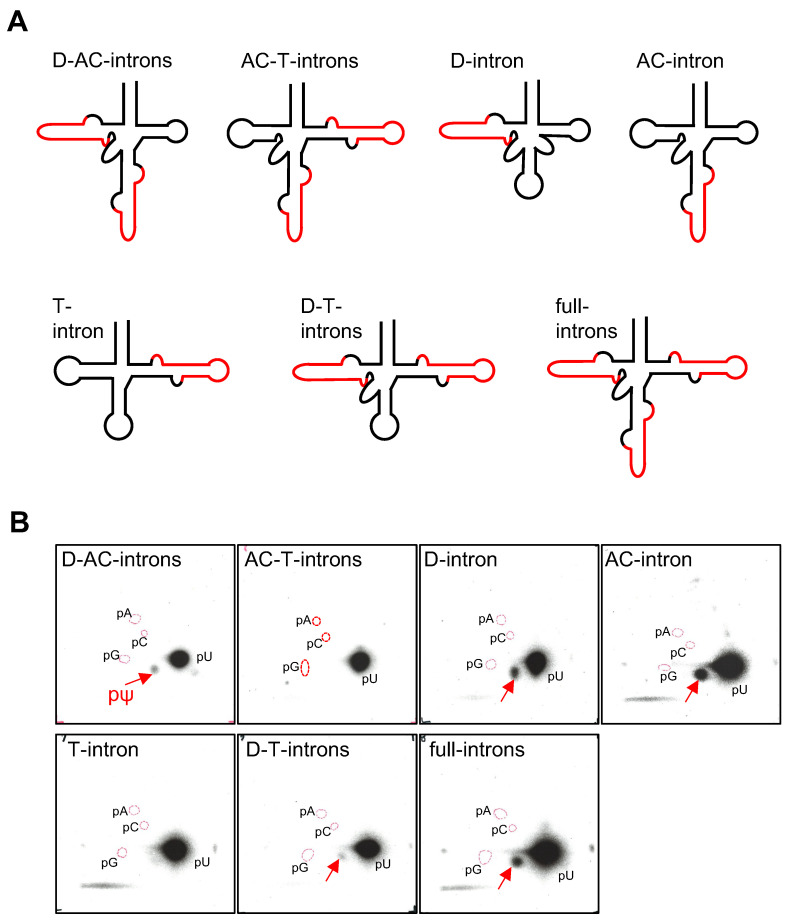
In vitro pseudouridylation activity of cmPus1 towards various pre-tRNA^Ile^(UAU) transcripts forms. (**A**) The secondary structures of pre-tRNAs^Ile^(UAU) used in the experiments are shown. Intron regions are indicated in red. (**B**) The ^32^P-internally labeled pre-tRNA^Ile^(UAU) variants were incubated with cmPus1, and Ψ formations analyzed by 2D-TLC. Spots derived from pΨ are indicated by red arrows. A pΨ spot was detected in pre-tRNA^Ile^(UAU) containing D-AC-, D-, AC-, D-T-, or full-intron(s).

The wild type pre-tRNA^Ile^(UAU) gene possesses three introns with bulge-helix-bulge (hBHBh’) structural motifs [25] in the D-stem (D-intron), anticodon-loop (AC-intron) and the T-loop (T-intron) (Figure 1) [23]. We prepared the seven presumptive types of pre- tRNA transcripts as shown in Figure 4A. These were tested for pseudouridylation by cmPus1. No pΨ spot was detected in the samples of the pre-tRNAs containing the AC-and T-introns (AC-T-introns) and the T-intron alone (Figure 4B). With the other five types of the pre-tRNAs [D-intron, AC-intron, D- and AC-intron (D-AC-introns), D-and T-introns (D-T-introns), and D-, AC-, and T-introns (full-introns)], Ψ modification(s) introduced by cmPus1 was detected (Figure 4B).

Subsequently, for the pre-tRNAs that accepted pseudouridylation, pre-tRNA mutant transcripts were prepared to allow the determination of the Ψ positions. The results are described in order as follows.

**Figure 5:**
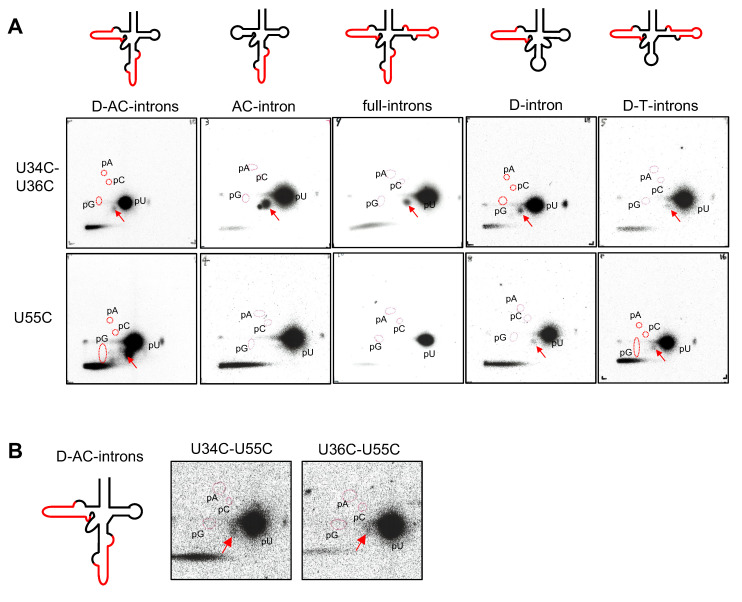
In vitro pseudouridylation activities of cmPus1 towards pre-tRNA^Ile^(UAU) mutants. Spots derived from pΨ are indicated by red arrows. (**A**) The α-^32^P-UTP-labeled pre-tRNA^Ile^(UAU) mutant was incubated with cmPus1, and Ψ formation analyzed by 2D-TLC. (**B**) In pre-tRNA^Ile^(UAU) containing D-AC-introns, the α-^32^P-UTP-labeled U34C-U55C or U36C-U55C mutant was incubated with cmPus1, and Ψ formation analyzed by 2D-TLC. Ψ modifications were detected in both mutants.

*Pre-tRNA^Ile^(UAU) Containing the D-AC-introns*: Ψ formation was detected in the pre-tRNA U34C-U36C mutant (Figure 5A). This predicted that the position of Ψ would be at position 55. With the pre-tRNA U55C sample treated with cmPus1, a pΨ spot appeared in the 2D-TLC analysis. Since the position(s) of Ψ was predicted to be at 34 and/or 36, pre-tRNA U34C-U55C and U36C-U55C mutants were prepared and analyzed. A very weak but detectable Ψ formation was observed for the pre-tRNA mutants (Figure 5B), whereas it was not detectable in the nearest neighbor analysis of the ATP-labeled pre-tRNA (Figure S4). Furthermore, with the pre-tRNA U34C-U35C-U55C mutant, no Ψ spot was detected (Figure S5). These results suggest that the cmPus1 accepts pre-tRNA containing the D-AC introns, and Ψ formation occurs mainly at position 55 and slightly at positions 34 and 36.

*Pre-tRNA^Ile^(UAU) containing the AC-intron or the full-introns:* Ψ formation was detectable in the pre-tRNA U34C-U36C mutants (Figure 5A). However, no pΨ spots appeared with the pre-tRNA U55C mutants (Figure 5A). Thus, cmPus1 forms Ψ only at position 55 in the pre-tRNAs containing the AC-intron or the full-introns. The result of the AC-intron was in agreement with that of pre-tRNA^Ile^(GAU) carrying the AC-intron (Figure S3).

*Pre-tRNA^Ile^(UAU) containing the D-intron or the D-T-introns:* Ψ formation was detectable in pre-tRNA U34C-U36C and U55C mutants (Figure 5A). In addition, the cmPus1 did not accept the pre-tRNA U34C-U35C-U55C mutant (Figure S5). Thus, the pre-tRNAs have the potential to have Ψ at positions 34 and/or 36 and 55. However, since the pre-tRNAs^Ile^(UAU) containing the D-intron and the D-T-introns were not detected in *C. merolae* RNA fraction [23], the intermediate pre-tRNAs might only barely be present in cells. 

Based on the experimental results, we conclude that the site specificity of cmPus1 is regulated by the presence of introns, at least in vitro. When all introns are absent, cmPus1 acts only on U55. When AC-intron is present, cmPus1 does not act on U34 and U36. Thus, AC-intron is a negative element for Ψ34 and Ψ36 modifications by cmPus1. However, this negative effect of AC-intron on Ψ34 and Ψ36 modifications is abolished by the existence of D-intron (results of D-AC-introns). Furthermore, the presence of T-intron negatively regulates the Ψ55 formation activity of cmPus1. However, this effect by T-intron is abolished by the presence of D-intron (results of full introns and D-T-introns). Moreover, U34 and U36 can be modified under the T- and D-intron regulation (results of D-T-introns). In addition, the presence of D-intron is essential for Ψ34 and Ψ36 modifications. 

### 2.5. Analysis of cmPus4 Mediated Pseudouridine Formation in Pre-tRNA^Ile^(UAU) In Vitro 

**Figure 6:**
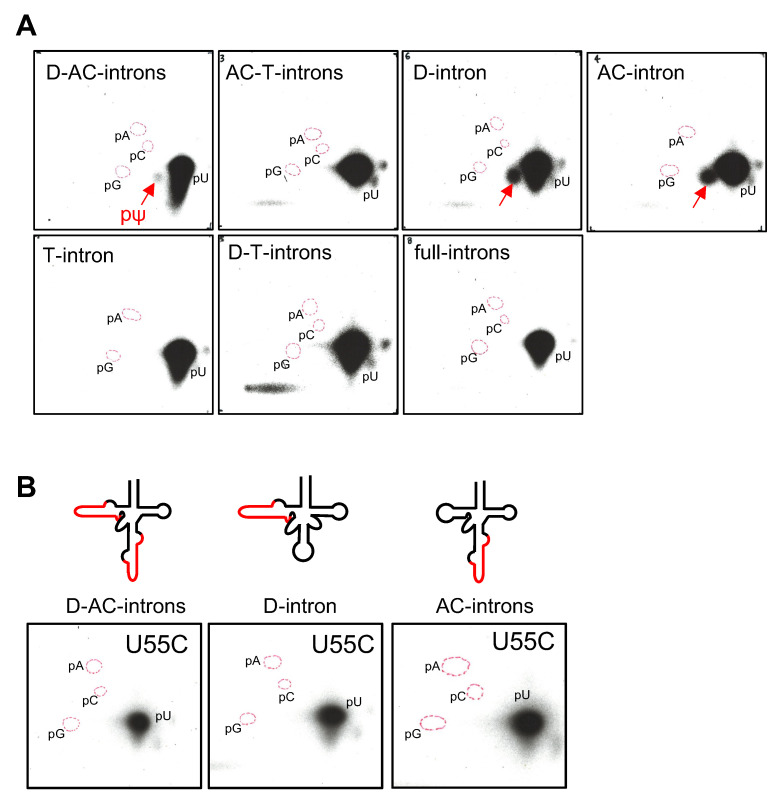
In vitro pseudouridylation activities of cmPus4 towards pre-tRNA^Ile^(UAU) and pre-tRNA^Ile^(UAU) mutants. Spots derived from pΨ are indicated by red arrows. (**A**) The α-^32^P-UTP^-^labeled pre-tRNA^Ile^(UAU) transcripts were incubated with cmPus4, and the Ψ formation was analyzed by 2D-TLC. A pΨ spot was detected in pre-tRNA^Ile^(UAU) containing D-AC-, D-, or AC-intron. (**B**) For the U55C mutant of pre-tRNA^Ile^(UAU) containing D-AC-, D-, or AC-intron, Ψ formation was analyzed by 2D-TLC. No Ψ spots appeared in any of the three samples.

As for cmPus1, the pseudouridine formation assay with cmPus4 was performed with pre-tRNA^Ile^(UAU) transcripts as shown in Figure 6A. cmPus4 accepted the pre-tRNAs^Ile^(UAU) containing the D-, AC-, and D-AC-introns (Figure 6A). Since aminoacyl-stem is close to position 55 in tRNA, the same experiment was performed for the intronless and intron-containing tRNAs with CCA attached to the 3′ end, considering the effect of the presence of the CCA terminal on the cmPus4 activity. These results were consistent with those against the tRNA variants without a CCA terminal (Figure S6). In the three pre-tRNA U55C mutants, no pΨ spots were detected in the three pre-tRNA U55C mutants (Figure 6B). Thus, the cmPus4 forms Ψ only at position 55 in the pre-tRNAs^Ile^(UAU) without T-intron, in addition to the intronless tRNA ^Ile^(UAU).

### 2.6. No RT-PCR Products Derived from Pre-tRNA^Ile^(UAU) Containing the D-Intron and D-T-Intron Were Detected

**Figure 7:**
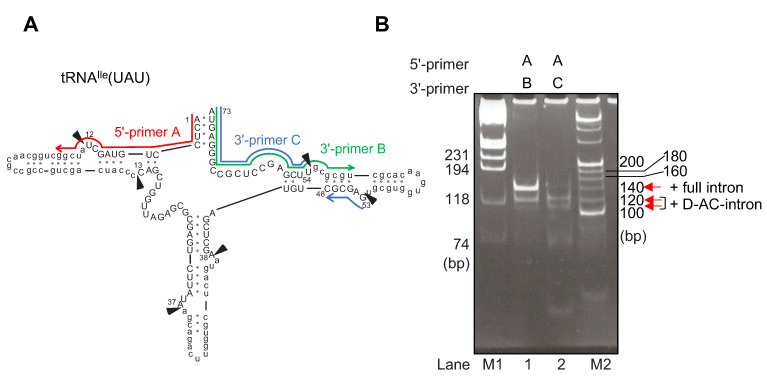
RT-PCR amplification of processing intermediates of *C. merolae* tRNA^Ile^(UAU). (**A**) The inferred secondary structures of the precursor of tRNA^Ile^(UAU) with three introns, one at 12/13 in the D-arm, one at 37/38 in the anticodon arm, and the third at 53/54 in the T-arm. Arrowheads indicate the positions to be processed. Intron sequences are shown in lowercase. (**B**) Processing intermediates of tRNA^Ile^(UAU) were amplified with 5′ -primer A (red arrow) and 3′ -primer B (green arrow) or 3′ -primer C (blue arrow) for RT-PCR. PCR products amplified from the cDNA of precursors and processing intermediates are indicated. The sizes of RT-PCR products are as follows: 146 bp for pre-tRNA^Ile^ (+ full intron); 126 bp for pre-tRNA^Ile^ (+D-AC-intron). Lane M1: DNA molecular marker (φX174/Hae III, NEB). Lane M2: DNA molecular marker (20 bp Ladder, Takara, Japan).

In a previous report by Soma et al. [23], it was demonstrated that no product was detected for an intermediate in which anticodon intron was removed before the D-arm intron and the T-arm intron on the RT-PCR analysis of *C. merolae* total RNA. Thus, it is supposed that the processing of D- and T-introns precedes that of anticodon intron [23]. We performed RT-PCR again using another primer set to detect the intermediates (Figure 7). The RT-PCR products derived from pre-tRNA^Ile^(UAU) variants containing D-AC introns and full-introns were detected. Annotation of each PCR product was based on sequencing analysis of gel purification of the bands followed by TA-cloning. In our experiment, RT-PCR products derived from either pre-tRNA^Ile^(UAU) containing the D-intron or D-T-intron could not be detected. Thus, these experimental results show that the Ψ modification patterns in the three pre-tRNAs (full-introns, D-AC-introns, and AC-intron) and the fully spliced intronless form reflect the physiological phenomena in the living cells. Two bands derived from D-AC-intron are indicated by the arrows in Figure 7 because these PCR products had the same sequence.

### 2.7. Position of Ψ in Native tRNA^Ile^(UAU)

**Figure 8:**
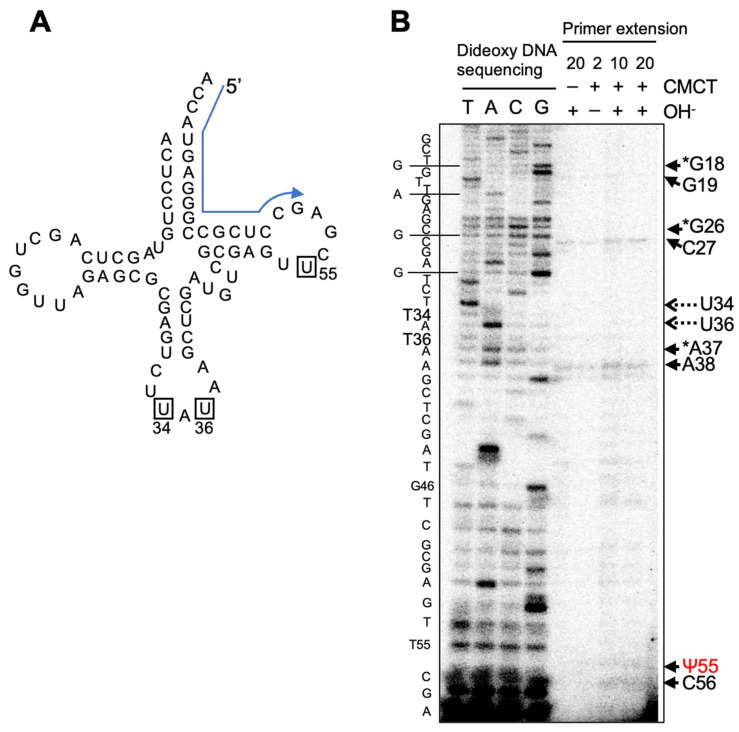
Detection of Ψ modifications in native tRNA^Ile^(UAU) by CMCT-RT. (**A**) The region complementary to a primer used for reverse transcription is marked with a blue arrow on the cloverleaf schematics. (**B**) Primer extensions were performed on class I tRNA from *C. merolae*. The class I tRNAs were treated with CMCT for 2, 10, and 20 min followed or not by alkaline (OH^−^) treatment (+ or −). Dideoxy DNA sequencing ladders (lanes T,A,C,G) were prepared with the same primers. The positions indicate with dashed arrows are U(T)34 and U(T)36. Black arrows indicate reverse transcription pause positions. Asterisks indicate modified nucleosides.

To clarify whether Ψ modifications are introduced at positions 34, 36, and 55 of the tRNA^Ile^(UAU) in the cell, a reverse transcription-based analysis was performed for an isolated class I tRNA from *C. merolae*. The primer was designed to be complementary from position 76 to position 59 in the tRNA (Figure 8A). The reverse transcription was paused at position 55, whereas no stop was detected at either position 34 or 36 (Figure 8B). Since reverse transcription also paused at positions 18, 26, and 37 regardless of CMCT treatment, it is considered that some modifications were introduced in the positions. In yeast tRNA, Gm18, *N*^2^-methylguanosine (m^2^G26) or *N*^2^, *N*^2^-dimethylguanosine (m^2^_2_G26), and *N*^6^-threonylcarbamoyladenosine (t^6^A37) are introduced in the positions [26]. As these modified nucleosides stop the reverse transcriptase reaction, Gm18, m^2^G26 (or m^2^_2_G26), and t^6^A37 may also be introduced in the tRNA^Ile^(UAU) of *C. merolae*.

## 3. Discussion

**Figure 9:**
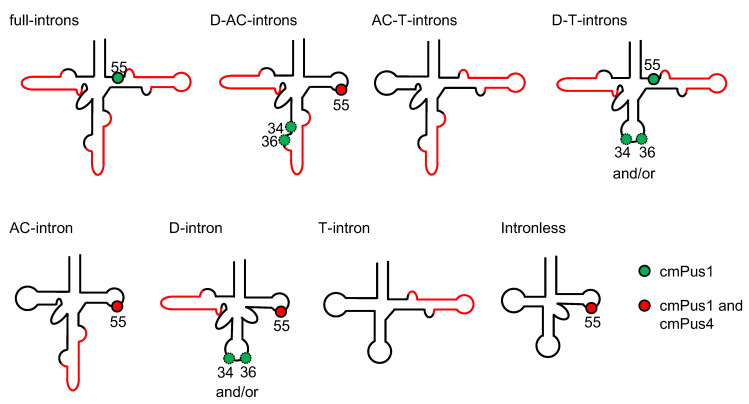
Possible Ψ positions in intron-less and -containing (pre)-tRNA^Ile^(UAU) variants introduced by cmPus1 and cmPus4. The Ψ positions resulting from treatment with cmPus1 or cmPus1 and cmPus4 are shown in green or red circles, respectively. Dotted circles indicate weak pseudouridylation activity. In the pre-tRNAs containing D-T- and D-intron, cmPus1 mediates Ψ modification at positions 34 and/or 36. Intermediates containing AC-T- or T-intron did not produce detectable Ψ conversion catalyzed by either cmPus1 or cmPus4.

In this study, we determined substrate and site specificities of cmPus1 and cmPus4 for pre-tRNA containing multiple introns using various pre-tRNA^Ile^(UAU) transcripts in vitro. A summary of the results is shown in Figure 9. cmPus1 can modify U34, U36, and U55 in some specific intron-containing pre-tRNA^Ile^(UAU) variants and can only form Ψ55 in intronless-tRNA^Ile^(UAU). The formation of Ψ34 and Ψ36 by cmPus1 occurs in very limited tRNA forms, and its activity is weak. The main pseudouridylation position of cmPus1 is U55, and the Ψ55 is formed except when tRNA^Ile^(UAU) has only T-intron with or without AC-intron. cmPus4 only modifies U55 but not in the presence of the T intron.

Yeast Pus1 catalyzes Ψ formation at positions 1, 26, 27, 28, 34, 35, 36, 65, and 67 in tRNAs [12,14,15,17,18] and at position 44 of U2 snRNA [27]. Therefore, Pus1 is a multiple-sites-specific enzyme and acts on multiple substrate RNAs. Yeast Pus1 is responsible for the Ψ formation at 34 and 36 of tRNA^Ile^(ΨAΨ), while the modification is dependent on the presence of an intron in the anticodon arm. We compared the amino acid sequences of cmPus1 and yeast Pus1 as well as human and mouse Pus1 (Figure S7). cmPus1 contains two Ψ synthase domains, each of which has 32% (96–276 aa) and 29% (510–607 aa) identity with yeast Pus1 (https://www.ncbi.nlm.nih.gov accessed on 1 August 2021). The identity is not extremely low. However, over 40 amino acid residues are present between domains III and IV in cmPus1, and the amino acid identity of domain IV is low (Figure S7). Our in vitro experimental results demonstrate that the site specificity of cmPus1 is regulated by the existence of the introns. Because we used purified cmPus1 and a pre-tRNA transcript throughout the experiments, the presence of other protein factors and guide RNA should not be considered [28]. When all introns are absent, cmPus1 acts only on U55. In the pre-tRNA^Ile^(UAU) containing AC-intron, cmPus1 does not act on U34 and U36. In addition, cmPus1 does not catalyze Ψ formation at least at position 27 from the experimental results using tRNA^Ile^(GAU) (Figure S3). This characteristic is completely different from yeast Pus1. The effect of D- and T-introns on the site specificity of cmPus1 is unprecedented. To understand this effect, structural studies of pre-tRNA and cmPus1 are required. Furthermore, the pre-tRNAs corresponding to D- and D-T-introns (Figure 7) were not detected in our current and previous [23] analyses of pre-tRNAs from *C. merolae* cells. Therefore, the Ψ formation in D- and D-T-introns are only in vitro experimental results.

In contrast to Pus1, Pus4 catalyzes Ψ formation only at position 55 and is a single-site-specific Ψ synthase. Ψ55 is commonly found in eubacteria, archaea, and eukaryotes [29,30]. The archaeal Ψ55 synthases are Cbf5 and Pus10 [31,32,33,34]. Since the archaeal Pus10 can convert U54 and U55 to Ψ modifications, Pus10 is a multiple-site-specific pseudouridine synthase. cmPus4 mediates Ψ modification at position 55 in intronless tRNA^Ile^(UAU) and pre-tRNA^Ile^(UAU) variants except when pre-tRNAs contain a T-intron. Although Ψ55 has been proposed to be introduced at a fairly early stage after the tRNA gene is transcribed [6], cmPus4 cannot form Ψ modification in pre-tRNA containing full introns. However, another pseudouridine synthase cmPus1 catalyzes Ψ55 formation in the pre-tRNA. 

The means of RNA recognition by Ψ synthetases allows the enzymes to be split into three classes. The first class consists of single-site-specific enzymes [16,35,36], the second consists of region-specific enzymes capable of modifying several neighboring positions [37,38], and the third consists of multiple-site and multiple-substrate-specific enzymes that modify distinct positions in different classes of RNAs [39]. In the current study, cmPus1 and cmPus4 are identified as belonging to the multiple-site-specific group and the single site-specific group, respectively. With regard to cmPus1, it is clear that U55 is converted to Ψ. Thus, the relationship between cmPus1 and cmPus4 is similar to that of yeast Pus1 and Pus7 [40,41]. Yeast Pus1 catalyzes Ψ formations at positions 34 and 36 in pre-tRNA^Ile^(UAU) and also at position 35 in pre-tRNA^Tyr^ in vitro. In addition, Ψ at position 35 in pre-tRNA^Tyr^ is also formed by the action of yeast Pus7 which also introduces Ψ at positions 35 of U2 snRNA [40,41]. 

Recently, in a study of *S. cerevisiae* mutant strains in which tRNA introns were removed from intron-containing tRNA genes, tRNA^Ile^(UAU) was analyzed. The tRNA^Ile^(UAU) intronless strain lacked the Ψ34 and Ψ36 modifications, and instead had a 5-carbamoylmethyluridine (ncm^5^U) modification at position 34 [42]. The six-Elongator protein subunit (Elp1-Elp6) complex is required for ncm^5^U synthesis [43], and the Elp complex is considered to catalyze the formation of ncm^5^U34 after intron splicing. In other words, Ψ is not required for the removal of the intron, and it is proposed that the presence of the intron prevents the introduction of the ncm^5^U modification [42]. Since the ncm^5^U34 allows for base-paring with G [44], tRNA^Ile^(UAU) carrying ncm^5^U34 may be used to decode the methionine AUG codon. In order to prevent misreading, Pus1 may form Ψ34 into tRNA^Ile^(UAU) containing an intron in yeast. Our experiments using UTP-labeled tRNA showed that Ψ formation at positions 34 and 36 by cmPus1 occurs very weakly in pre-tRNA^Ile^(UAU) transcript containing the D-AC-introns. Ψ34 and Ψ36 also were undetectable in the nearest neighbor experiment using ATP-labeled tRNA. In a reverse transcription-based analysis for the native tRNA^Ile^(UAU), no pauses of the reverse transcriptase were observed at positions 34 and 36. These results indicate that in the native tRNA^Ile^(UAU), Ψ modification might be absent or only partially introduced at positions 34 and/or 36. The experimental result using RNA from *C. merolae* cells is consistent with that pseudouridylation activity for U34 and U36 by cmPus1 was very weak in the in vitro experiment. We also suggest that the pseudouridylation in the pre-tRNA^Ile^(UAU) is unlikely to be a trigger for the AC-intron cleavage. This study clarifies the relationship between introns and pseudouridylation in tRNA^Ile^(UAU) which harbors multiple introns and provides important insights into the processing system of tRNA in *C. merolae*.

## 4. Materials and Methods

### 4.1. Cell Culture 

*C. merolae* 10D cells were grown on Allen’s medium [45] with shaking under continuous light (50 W/m^2^) at 42 °C for 1 week. *Escherichia coli* was cultured in LB medium or MM9 medium (42 mM Na_2_HPO_4_, 22 mM KH_2_PO_4_, 34 mM NaCl, 20 mM glucose, 2 mM MgSO_4_, 0.1 mM CaCl_2_, 0.3% casamino acid). 

### 4.2. Construction of the Expression Vector

The CYME_CMH251C and CYME_CMF022C genes from *C. merolae* have been annotated as “similar to tRNA-pseudouridine synthase I” and “tRNA pseudouridine 55 synthase”, respectively [20]. The CYME_CMH251C gene and CYME_CMF022C gene products were named cmPus1 and cmPus4 to represent candidate homologs of yeast Pus1 and Pus4, respectively. The *C. merolae* genomic DNA was extracted using the cetyltrimethylammonium bromide (CTAB) extraction method [46]. The two genes were amplified by PCR from the genomic DNA of *C. merolae* using KOD Fx neo (Toyobo, Osaka, Japan) and primers (Table S1). The obtained PCR product was assembled into a pET22b PCR fragment using NEBuilder HiFi assembly (New England BioLabs, United States). The resulting expression plasmids were designated as pET22b-cmPus1-His and pET22b-His-cmPus4.

### 4.3. Preparation of the cmPus1 and cmPus4 Gene Product

Recombinant cmPus1 was expressed on *E. coli* Rosetta 2 (DE3) at 25 °C for 19 h with induction by addition of 1 mM (final concentration) isopropyl-β-D-1-thiogalactopyranoside. Harvested cells were suspended in a buffer A [50 mM Tris-HCl (pH 8.0), 150 mM NaCl, 1 mM DTT] containing protease inhibitor (Pierce, United States) and then disrupted with an ultrasonic processor (Sonics and Materials, Inc., Newtown, CT, USA). The supernatant after centrifugation was loaded onto a Ni Sepharose High Performance (GE Healthcare, Chicago, IL, USA) column equilibrated with buffer A and eluted with a stepwise gradient of 20, 50, 150, or 250 mM imidazole in buffer A. The fraction containing cmPus1 was eluted with the 150 mM imidazole buffer and then dialyzed against buffer B [20 mM Tris-HCl (pH 8.0), 1 mM MgCl_2_, 1 mM DTT, 10% glycerol]. After dialysis, the sample was loaded onto a Q Sepharose Fast Flow (GE Healthcare, United States) column equilibrated with buffer B and eluted with a stepwise gradient of 50, 150, 250, 350, or 500 mM NaCl in buffer B. cmPus1 was present in the 250 mM NaCl elution sample. 

*E. coli* Rosetta 2 (DE3) transformed with the pET22b-His-pus4 was pre-cultured at 37 °C for 16 h, and then the pre-culture medium (50 mL) was seeded into MM9 medium (1000 mL) at 37 °C for 3.5 h (~0.8 OD_590_). The expression of recombinant cmPus4 was performed by the addition of 1 mM (final concentration) isopropyl-β-D-1-thiogalactopyranoside and then further cultured at 37 °C for 2 h. The cmPus4 protein was purified by the same protocol for the cmPus1 purification described above. Briefly, the cmPus4 fraction was prepared using a Ni Sepharose High-Performance column. The cmPus4 fraction eluted with buffer A containing 20–150 mM imidazole was further purified using a Q Sepharose Fast Flow column. cmPus4 was eluted in buffer B containing 500 mM NaCl, and the fraction was dialyzed against buffer C [50 mM Tris-HCl (pH 8.0), 5 mM MgCl_2,_ 6 mM β-mercaptoethanol, 50 mM NaCl]. 

The fraction containing cmPus1 or cmPus4 was concentrated using an Amicon Ultra-15 (Millipore, United States) and then added to an equivalent volume of 100% glycerol and stored at −30 °C. Protein concentration was measured with a Bio-Rad protein assay kit (Quick Start^TM^ Bradford Dye Reagent, Bio-Rad Laboratories, United States) and analyzed by 12% SDS-PAGE.

### 4.4. tRNA Preparation

Template DNAs for T7 RNA polymerase-mediated transcription reactions for the preparation of *C. merolae* tRNA^Ile^(UAU) variants and tRNA^Ile^(GAU) were prepared using the primers (Table S1). To prepare a transcriptional template for pre-tRNA^Ile^(UAU) containing full introns, *C. merolae* genome was used as a PCR template. Internally labeled tRNA^Ile^(GAU) and tRNA^Ile^(UAU) variants were prepared by transcription with α-^32^P-UTP, α-^32^P-CTP, or α-^32^P-ATP (PerkinElmer, Waltham, MA, USA). The tRNA transcripts were purified using a Q Sepharose Fast Flow column and then analyzed by 10% PAGE (7 M urea). The ^32^P internal labels in the transcripts were monitored using a Typhoon FLA 7000 imager (GE Healthcare, United States). 

### 4.5. Detection of Pseudouridine Formation by Two-Dimensional Thin Layer Chromatography (2D-TLC)

The internally labeled tRNA transcript (containing ^32^P at around 3000 cpm) and 150 ng of the purified cmPus1 or cmPus4 in 50-µL buffer D (100 mM Tris-HCl (pH 8.0), 100 mM ammonium acetate, 5 mM MgCl_2_, 2 mM DTT, 0.1 mM EDTA) were incubated at 37 °C for 1 or 2 h. The tRNA was extracted with phenol-chloroform and then recovered by ethanol precipitation. 0.8 A_260_units total RNA from *Lactobacillus plantarum* was added before ethanol precipitation. The tRNA pellet was dissolved and digested overnight at 37 °C with 3 µL (1 unit) nuclease P1 (Wako, Tokyo, Japan) in 50 mM ammonium acetate (pH 5.0) or RNase T2 in 20 mM ammonium acetate (pH 5.0). Nuclease P1 or RNase T2 was used for UTP- or ATP- and CTP-labeled tRNA transcripts, respectively. An aliquot of the sample was spotted onto a thin layer plate (Merck, Darmstadt, Germany, TLC Cellulose F; 10 cm × 10 cm) and separated as described previously [47]. The ^32^P-labeled nucleotides were monitored using a Typhoon FLA 7000 imager (GE Healthcare, United States). Standard nucleotides were marked by UV_260nm_ irradiation.

### 4.6. RNA Isolation

Small RNA fraction was extracted using ISOGEN II (Nippon Gene, Tokyo, Japan) according to the manufacturer’s manual. 140 µg of small RNA was isolated from 1 g of *C. merolae* 10D cells. Class I tRNA fraction was further purified by 10% PAGE (7 M urea).

### 4.7. CMCT Modification and Reverse Transcription 

To detect the Ψ positions in tRNA, a combination of chemical modification and reverse transcription was performed. The tRNA^Ile^(UAU) transcript was incubated with the recombinant cmPus1 or cmPus4 as described above. The tRNAs were extracted with phenol-chloroform and then collected by ethanol precipitation. The Ψ modifications of the tRNA transcripts and isolated class I tRNA were chemically modified using a CMCT according to the literature [24]. The positions of CMCT-chemical modifications were determined by reverse transcription and DNA sequencing. Reverse transcriptions were performed using 5′-^32^P-labeled primers (Table S1), 1 mM dNTPs, and ReverTraAce (Toyobo, Japan) as described [48]. 

### 4.8. Reverse Transcription Polymerase Chain Reaction (RT-PCR) and DNA Sequencing

The analysis of RT-PCR and DNA sequencing were performed following previously described methods [21,23].

## Data Availability

Not applicable.

## Supplementary Materials

The following supporting information can be downloaded at: https://www.mdpi.com/article/10.3390/ijms232012058/s1, Reference [49] are cited in the supplementary materials.
