# Supplementary material for: Intron-Dependent or Independent Pseudouridylation of Precursor tRNA Containing Atypical Introns in Cyanidioschyzon merolae
Source: Int J Mol Sci. 2022 Oct 11;23(20):12058. doi: 10.3390/ijms232012058 (PMC9602550; doi:10.3390/ijms232012058)
Supplement: Supplementary file 1 [file ijms-23-12058-s001.zip › ijms-1913684-supplementary.pdf]

Figure S1

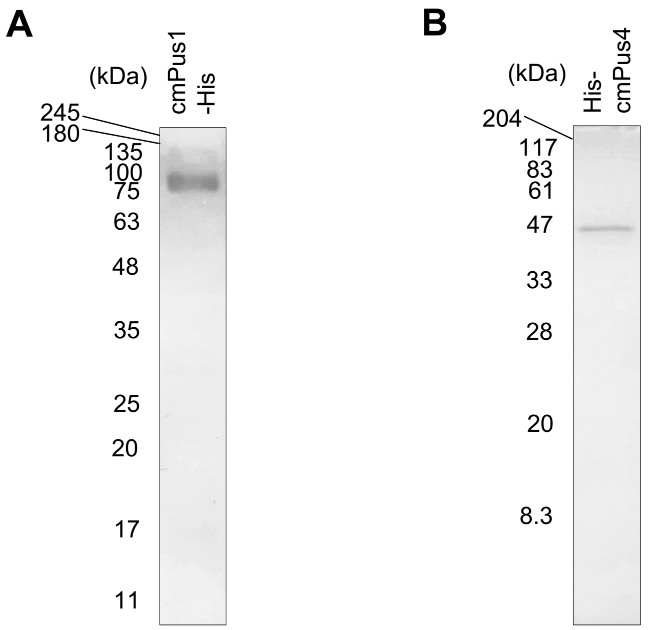

Figure S1. Purified recombinant cmPus1 and cmPus4. Purified cmPus1(A) and cmPus4 (B) were analyzed by 12% SDS-PAGE and detected by Coomassie Brilliant Blue staining.

Figure S2

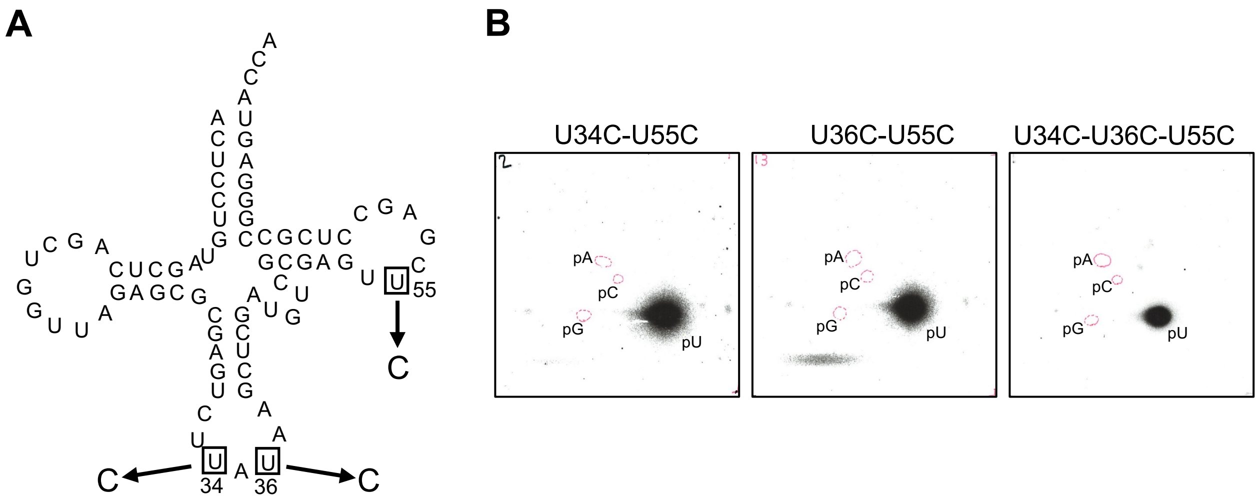

Figure S2. Pseudouridylation activities of cmPus1 for intronless tRNA^{Ile}(UAU) mutants. (A) Mutation positions are shown in a cloverleaf structure of intronless tRNA^{Ile}(UAU) in *C. merolae*. (B) The ^{32}P-UTP labeled pre-tRNA^{Ile}(UAU) of U34C-U55C, U36C-U55C, or U34C-U36C-U55C mutant was incubated with cmPus1, and Ψ formation was analyzed by 2D-TLC. No Ψ spots were detected in the three samples.

Figure S3

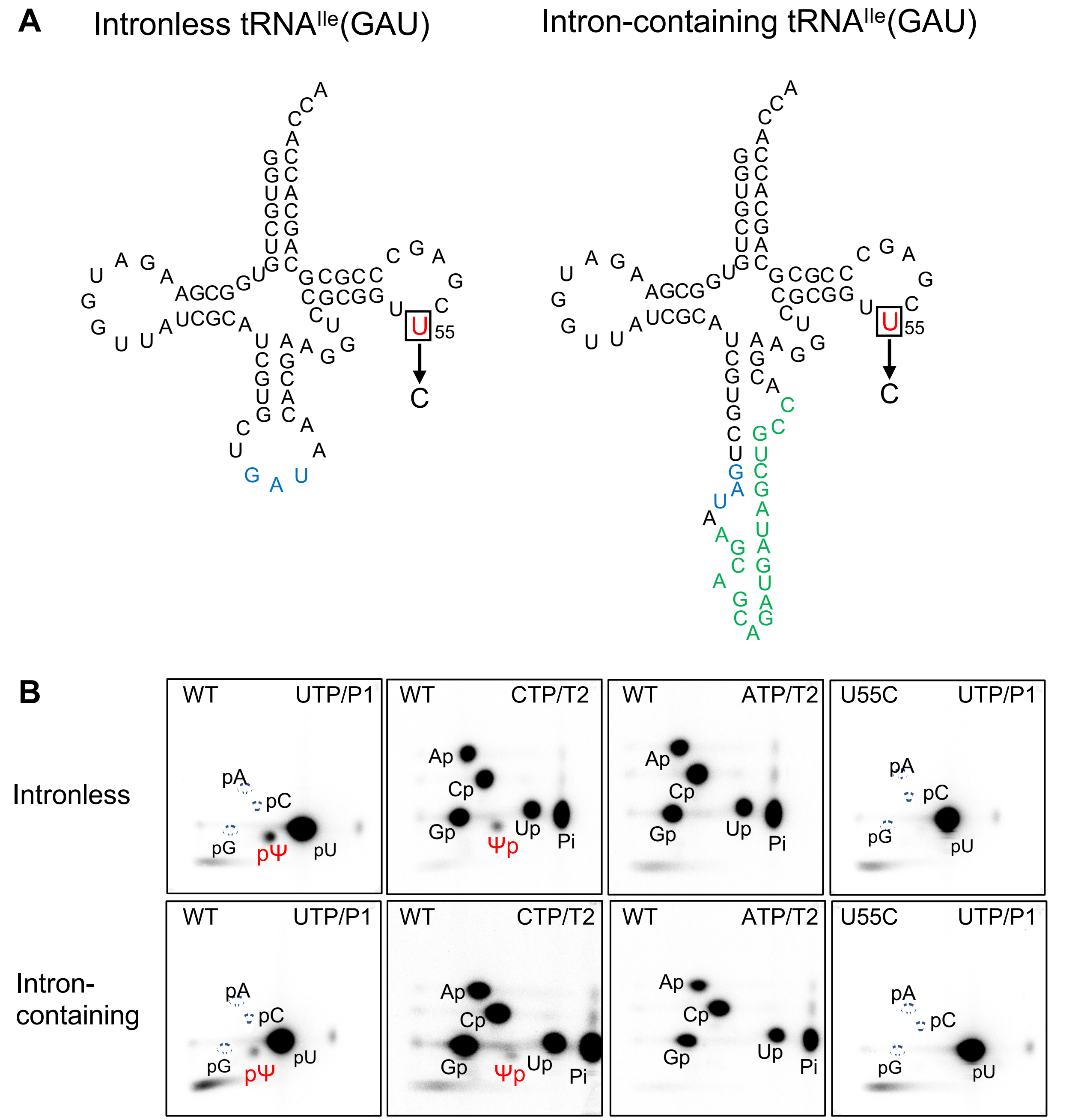

Figure S3. Pseudouridylation activities of cmPus1 for intron -less and -containing tRNA^{Ile}(GAU). (A) Cloverleaf structures of intron -less (left) and -containing (right) tRNA^{Ile}(GAU) are shown. Anticodon regions and bases of the intron are shown in blue and green letters, respectively. Mutation positions are shown in the cloverleaf structures. (B) UTP, CTP, and ATP refer to ^{32}P-labeled nucleotide incorporated during the transcription reaction. The ^{32}P-internally labeled intron -less or -containing tRNA^{Ile}(GAU) variant was incubated with cmPus1. After the incubation, the RNA was digested by nuclease P1 (P1) or RNase T2 (T2) and the resulting nucleotides were analyzed by 2D-TLC.

Figure S4

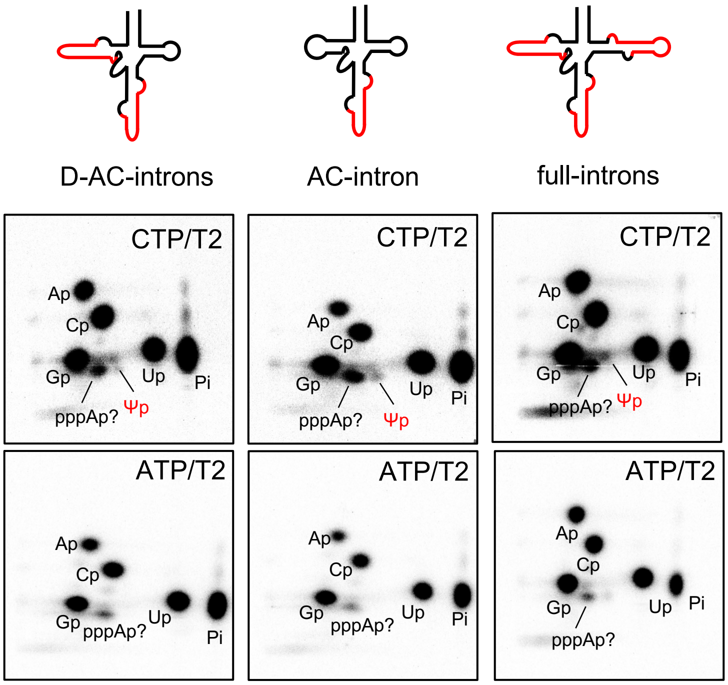

Figure S4. 2D-TLC analysis of the cmPus1 activities for pre-tRNA^{Ile}(UAU) transcripts. CTP or ATP labeled pre-tRNA^{Ile}(UAU) variants were incubated with cmPus1, and then the RNAs were digested by RNase T2. The resulting nucleotides were analyzed by 2D-TLC.

Figure S5

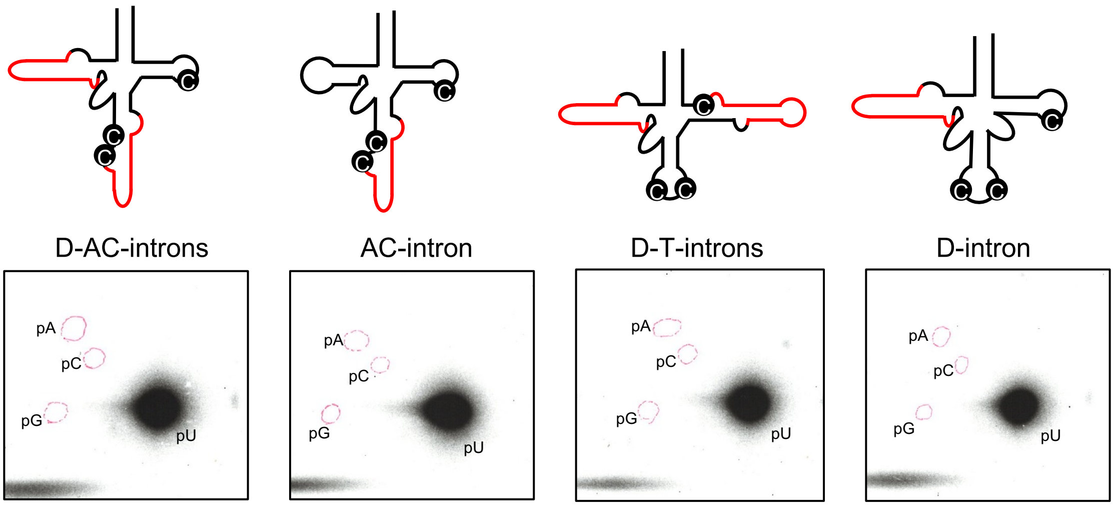

Figure S5. Pseudouridylation activities of cmPus1 for pre-tRNA^{Ile}(UAU) triple mutants. Since cmPus1 mediated formation of Ψ modification(s) in mutant (U34C-U36C and U55C) pre-tRNA^{Ile}(UAU) containing D-AC, AC-, D-T, and D-introns, the activity was also analyzed in triple mutants of the pre-tRNAs U34C-U35C-U55C. No Ψ spots were detected in the four samples.

Figure S6

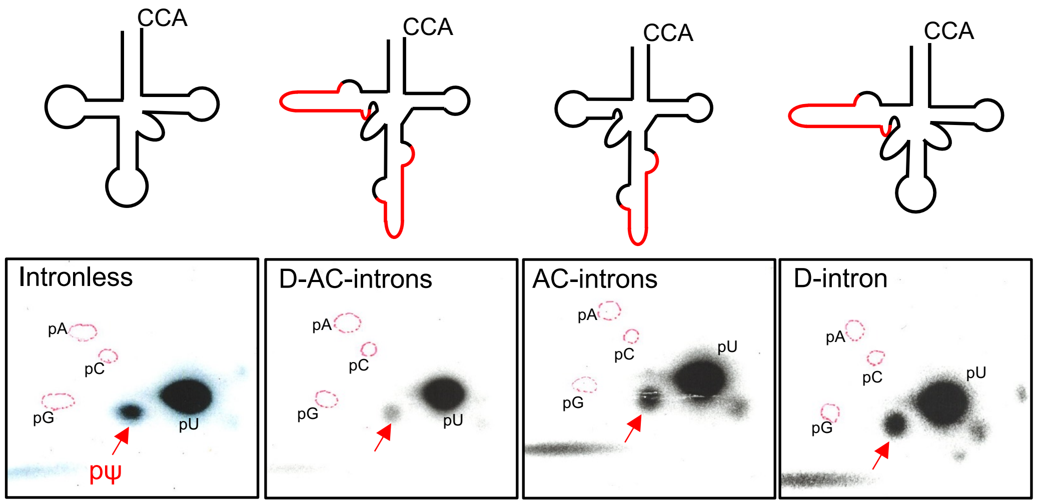

Figure S6. Pseudouridylation activities of cmPus4 for intronless tRNA^{Ile}(UAU) and intron-containing pre-tRNA^{Ile}(UAU) variants. When CCA was attached to the 3' end of the tRNA variants, intronless tRNA^{Ile}(UAU) and pre-tRNAs^{Ile}(UAU) containing D-, AC-, and D-AC-introns were also pseudouridylated by cmPus4.

Figure S7

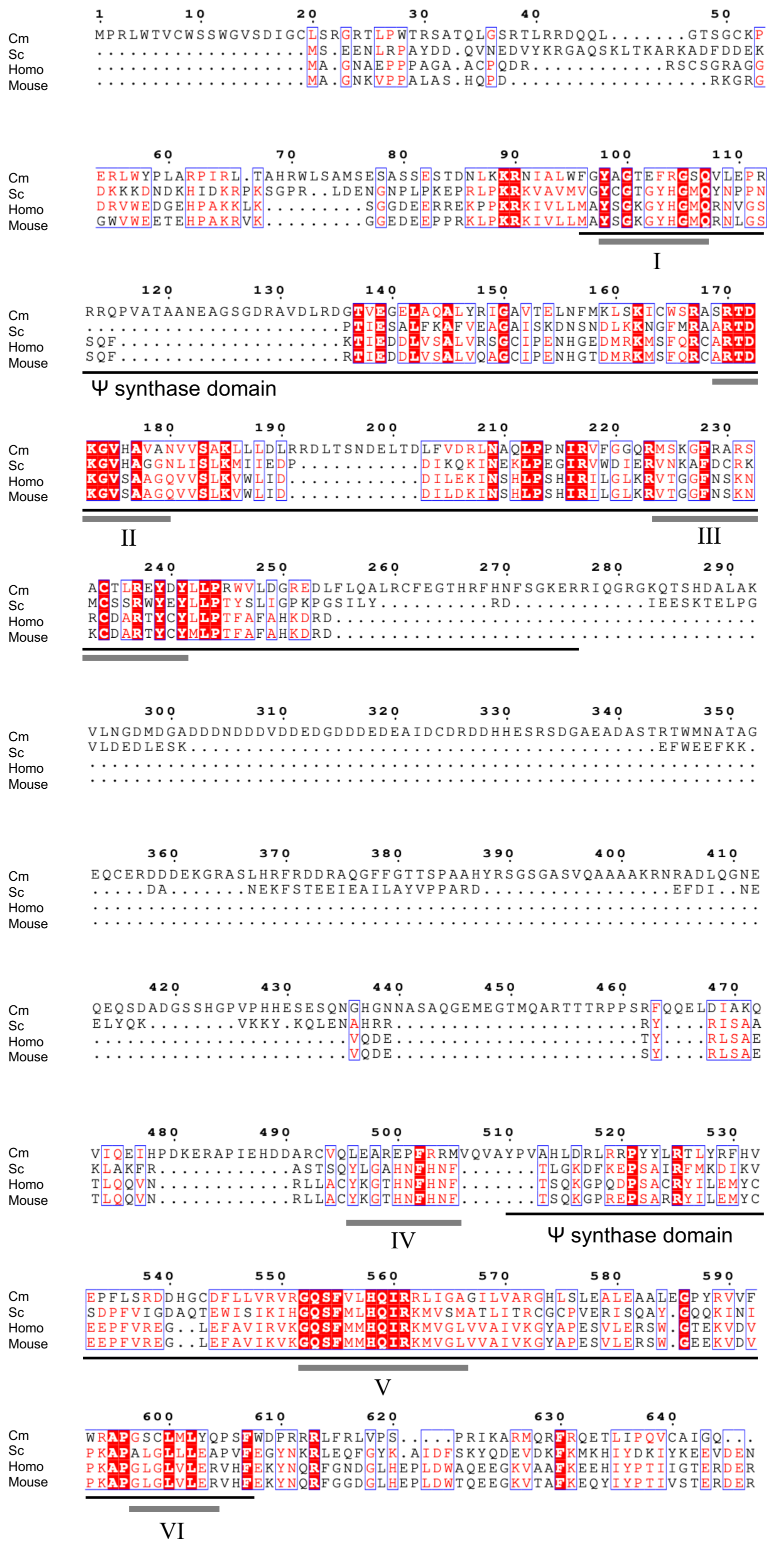
Cm
Sc
Homo
Mouse
I
Ψ synthase domain
II
III
IV
Ψ synthase domain
V
VI

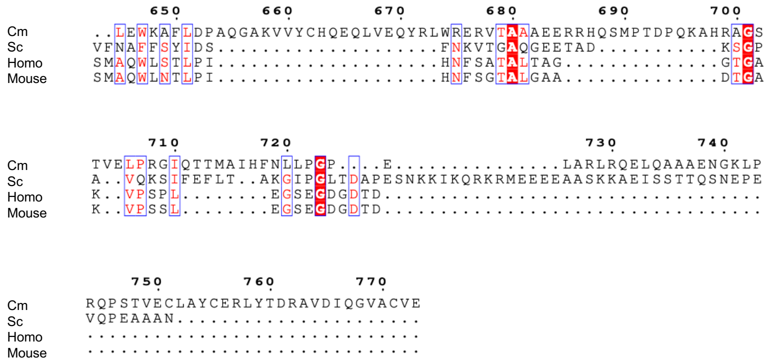

Figure S7. Sequence alignment of cmPus1 with the yeast, human, and mouse homologous. The numbering of residues is based on cmPus1. The domain numbering of I-VI is based on reference (Chen and Patton 1999).

Supplementary reference

Chen J, Patton JR. 1999. Cloning and characterization of a mammalian pseudouridine synthase. *RNA*, **5**, 409–419. doi: 10.1017/s1355838299981591
